# Ultrasonography in diagnosis and analysis of chronic pain following anterior open inguinal herniorrhaphy

**DOI:** 10.1186/s12893-018-0361-z

**Published:** 2018-05-22

**Authors:** ZY Qiu, Y Chen, JX Tang, L Chen

**Affiliations:** 1 Ultrasound Department, Huadong Hospital Affiliated to Fudan University, 221 West Yanan Road, Shanghai, 200041 China; 2 Department of General Surgery, Huadong Hospital Affiliated to Fudan University, 221 West Yanan Road, Shanghai, 200041 China

**Keywords:** Ultrasonography, Anterior open inguinal herniorrhaphy, Chronic pain

## Abstract

**Background:**

Chronic pain as a complication following inguinal herniorrhaphy has attracted increasing attention in recent years. There is evidence that the chronic pain seriously affects patients’ quality of life. However, there are few imaging studies and diagnostic techniques of the chronic pain. The aim of this study is to explore the etiology and to analysis ultrasonographic imaging description of chronic pain following anterior open inguinal herniorrhaphy.

**Methods:**

One hundred fifty two patients with the chronic pain following anterior open inguinal herniorrhaphy were performed by ultrasonography to identify the main causes of postoperative chronic pain. Positive ultrasonic diagnoses were confirmed to be correct by the pain relieved when the patients underwent re-operation and other clinical operations. Positive diagnoses which appeared simultaneously were grouped for pairwise comparisons.

**Results:**

Two hundred sixteen positive ultrasonic diagnoses, 12 categories of postoperative chronic pain were found. They were encapsulated effusion, scrotal wall edema, testitis, hydrocele testis, restricted motion of spermatic cord at the reconstructed deep inguinal ring, varicocele, scar sutured into pubic tubercle, shrinking mesh, accumulational mesh or mesh plug, recurrent hernia, cyst of spermatic cord and epididymal cyst. In the pairwise comparison groups, encapsulated effusion with scrotal wall edema, varicocele with restricted motion of spermatic cord at the reconstructed deep inguinal ring, and shrinking mesh with recurrent hernia had significant differences in each intragroup comparisons(*P* < 0.05).

**Conclusions:**

Ultrasonography provieds important value in the diagnosis of chronic pain following anterior open inguinal herniorrhaphy. Some positive diagnoses occur simultaneously, which is necessary for doctors to consider comprehensively.

## Background

It was pointed out in *Guidelines for diagnosis and treatment of adult inguinal hernia (2014 edition)* [ 1] that almost all cases of inguinal hernia are treated surgically, and anterior open inguinal herniorrhaphy is considered to be the most common surgical procedure. Also, the chronic pain is a late complication of the surgery. In the 1980s, the chronic pain was reported [2–4] to be a rare and occasional postoperative complication, and the assessment of the surgery was limited to the postoperative acute phase. Nevertheless, studies in recent years have found that the incidence of chronic pain following inguinal herniorrhaphy is approximately 54%, which is far higher than that reported previously. Furthermore, up to 50% of patients experience the chronic pain for more than 1 year, which seriously affects the patients’ quality of life [5–7]. The chronic pain has gradually become one of the long-term assessment indicators of the herniorrhaphy. Non-invasive, nonradiative and easily performed, ultrasound technique has been proposed as a valuable imaging method in examining patients before and after the inguinal herniorrhaphy [8].

The aim of our study was to explore the etiology and analyze positive findings of chronic pain after anterior open inguinal herniorrhaphy, thereby to assist in making treatment protocols in clinics.

## Methods

### Subjects

We conducted a prospectively, observational study in a single hernia center of Huadong Hospital, between August 2009 and May 2014. 539 patients who felt uncomfortable after their first herniorrhaphy were performed by ultrasonography in our ultrasound department. 152 patients who met the inclusion criteria agreed to participate in this study and signed the informed consent. This study was approved by Fudan University Ethics Committee. No statistical power calculation was conducted prior to the study. The sample size was based on the previous literature experience about the chronic pain review.

The inclusion criteria: the patients had a history of anterior open inguinal herniorrhaphy, during which artificial material was patched in preperitoneal space or the back wall of the inguinal canal was reconstructed by suturing. According to the definition of chronic pain by International Association of the Study of Pain (IASP) [9], cases with “*pain lasting for 3 months or more*” were included. Persistent chronic pain with or without local swell in the operative region or the ipsilateral scrotum was the clinical symptom of the disease. Sometimes the symptom could be relieved by holding up the scrotum.

The patients had laparoscope totally extraperitoneal repair(TEP) and transabdominal preperitoneal repair(TAPP) were excluded. And the patients younger than 16 years were excluded.

The longest course of postoperative chronic pain in this study was ten years, and the shortest course was 3 months. Among 31 patients who had a history of bilateral herniorrhaphy, 12 patients presented bilateral chronic pain. Ultimately, a total of 164 chronic pain sites met the inclusion criteria, included 157 sites with artificial mesh, 2 sites with mesh plug, and 5 sites with suture repair.

### Instruments and methods

The Siemens ACUSON S2000™ color doppler ultrasound instrument, equipped with ultrasonic volume auto-scan(UVAS) and a high-frequency linear transducer ranging from 9 to 12 MHZ, were performed to scan the herniorrhaphic incisions and chronic pain sites in patients. The following parameters of UVAS were set: a volume image of 15.4 cm (length) × 16.8 cm (width) × 6.0 cm (depth) captured with a minimum slice of 0.5 mm and 250 to 400 images were collected during a single scan. The axial sequence images were automatically transferred to the ultrasonic workstation for data analysis and processing. The patients were in supine position or standing position as needed for ultrasound scanning, with the scrotums simultaneously scanned in male patients. The location, shape, and spatial structure of the mesh, peritoneum, spermatic cords, etc., were observed, with the goal of achieving ultrasonographic diagnosis of the etiology of the postoperative chronic pain.

All ultrasonographic results were confirmed by the re-operation, the puncture or other clinical operations.

Cases with two or more positive diagnoses were pairwise grouped for multiple comparisons.

### Statistical analysis

Statistical analyses were performed by STATA10 software. Values were presented as mean ± standard deviation for normally distributed variables. Positive ultrasonic diagnoses in the pairwise groups compared using a chi-square test. Statistical significance threshold was considered α = 0.05.

## Results

The mean age in the study was 66.10 years(± 13.82SD), ranging from 22 to 93 years old. No statistical difference was observed between genders in 93 men and 59 women (χ^2^ = 0.35, *P* > 0.05).

### Ultrasonic diagnosis of chronic pain sites following anterior open inguinal herniorrhaphy

Among 164 chronic pain sites, 121 sites showed positive findings on ultrasonography and no ultrasonic abnormality was found in 43 sites.

**Fig. 1:**
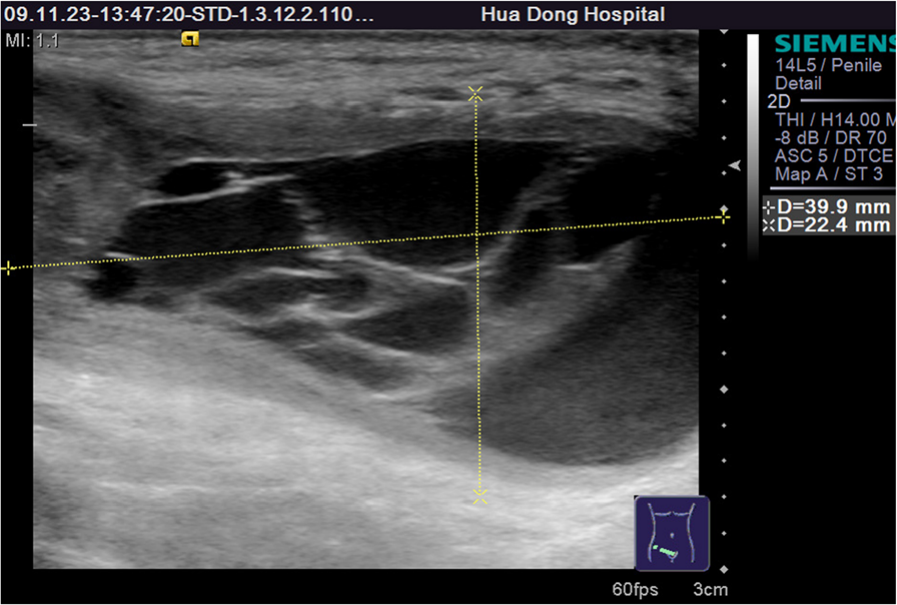
The seroma showed in the ultrasonography. The multilocular seroma was visible in front of the mesh

**Fig. 2:**
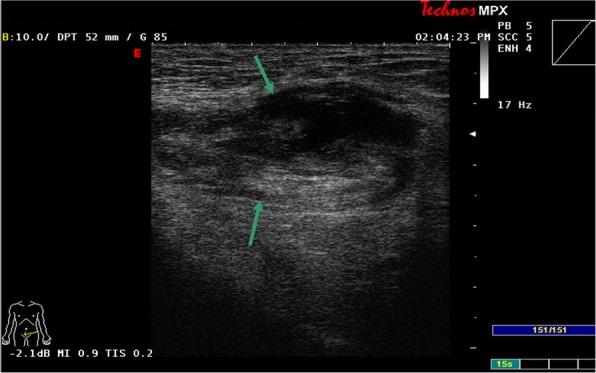
The hematoma showed in the ultrasonography. Arrows referred to the old hematoma

**Fig. 3:**
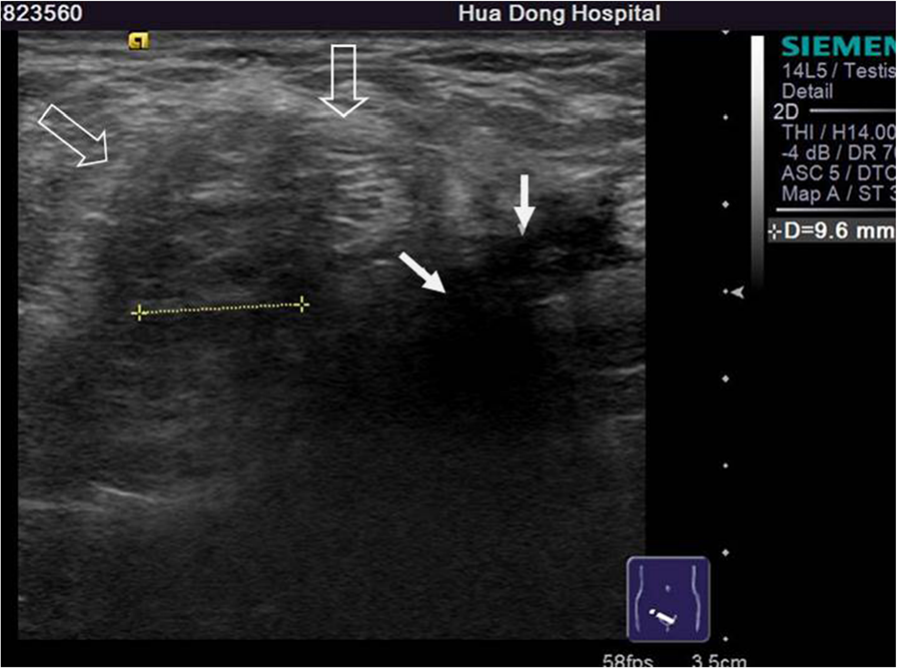
The recurrent hernia and the shrinking mesh showed in the ultrasonography. Hollow arrows referred to the recurrent hernia and solid arrows referred to the shrinking mesh. The measurement of straight line referred to the diameter of the perito neal defect of the recurrent hernia

**Fig. 4:**
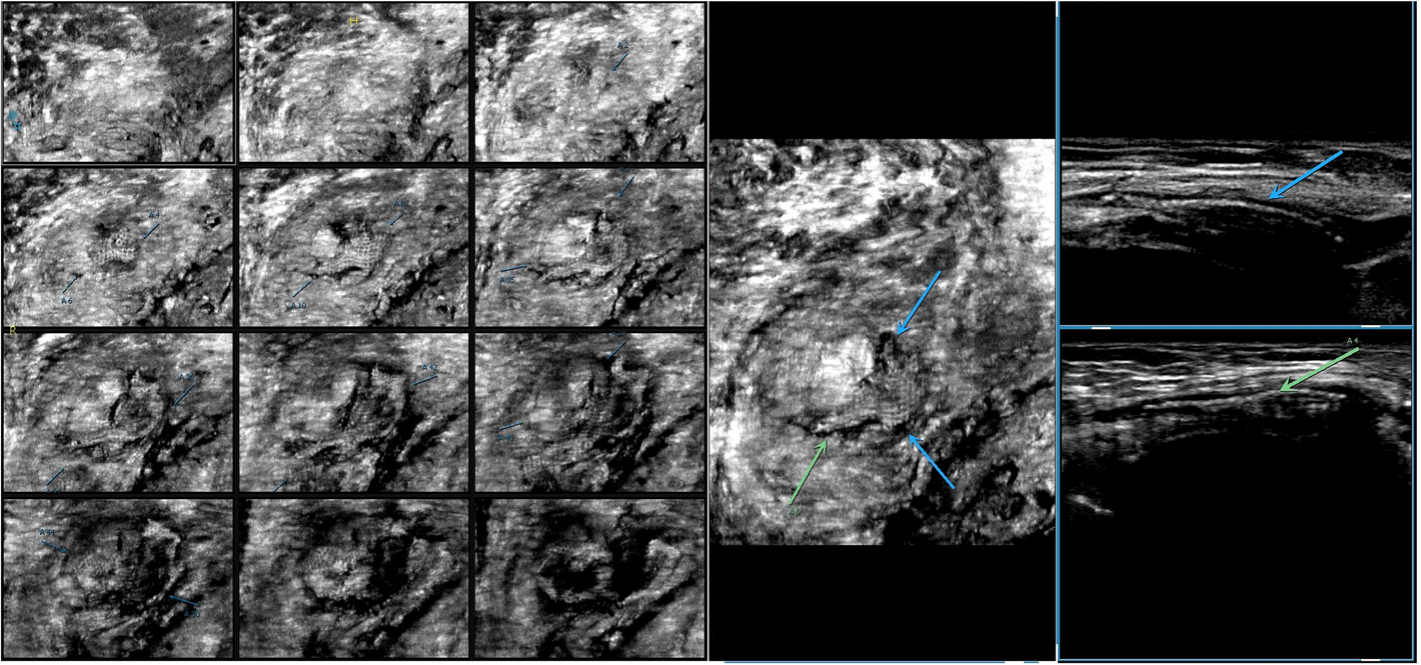
The shrinking mesh showed in the ultrasonography. The shrinking mesh was showed on coronal consecutive image and 3D display. Arrows referred to the shrinking mesh

**Fig. 5:**
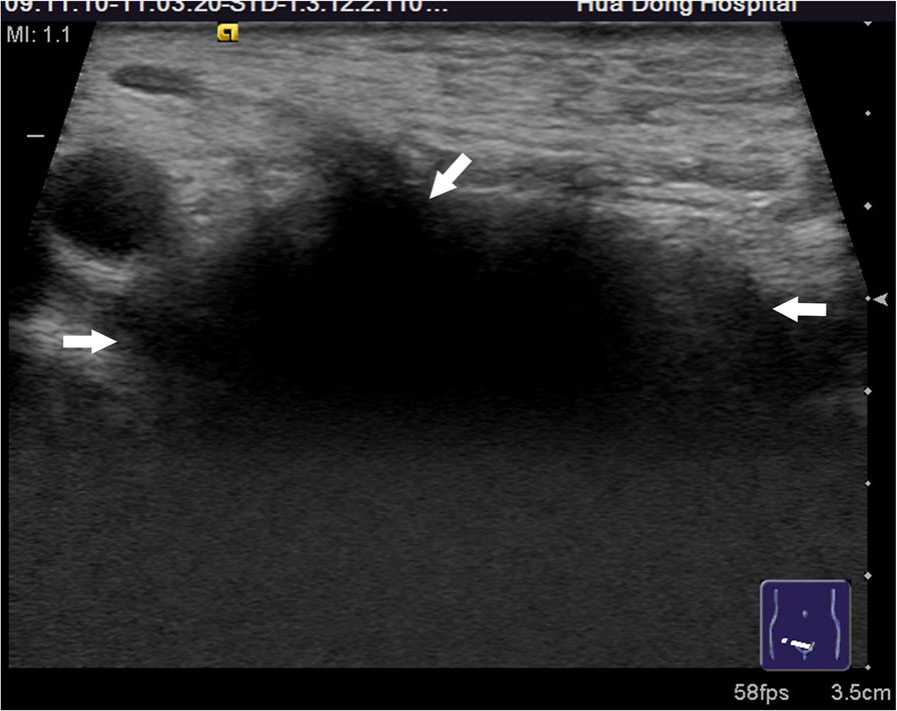
Accumulational meshes showed in the ultrasonography

**Fig. 6:**
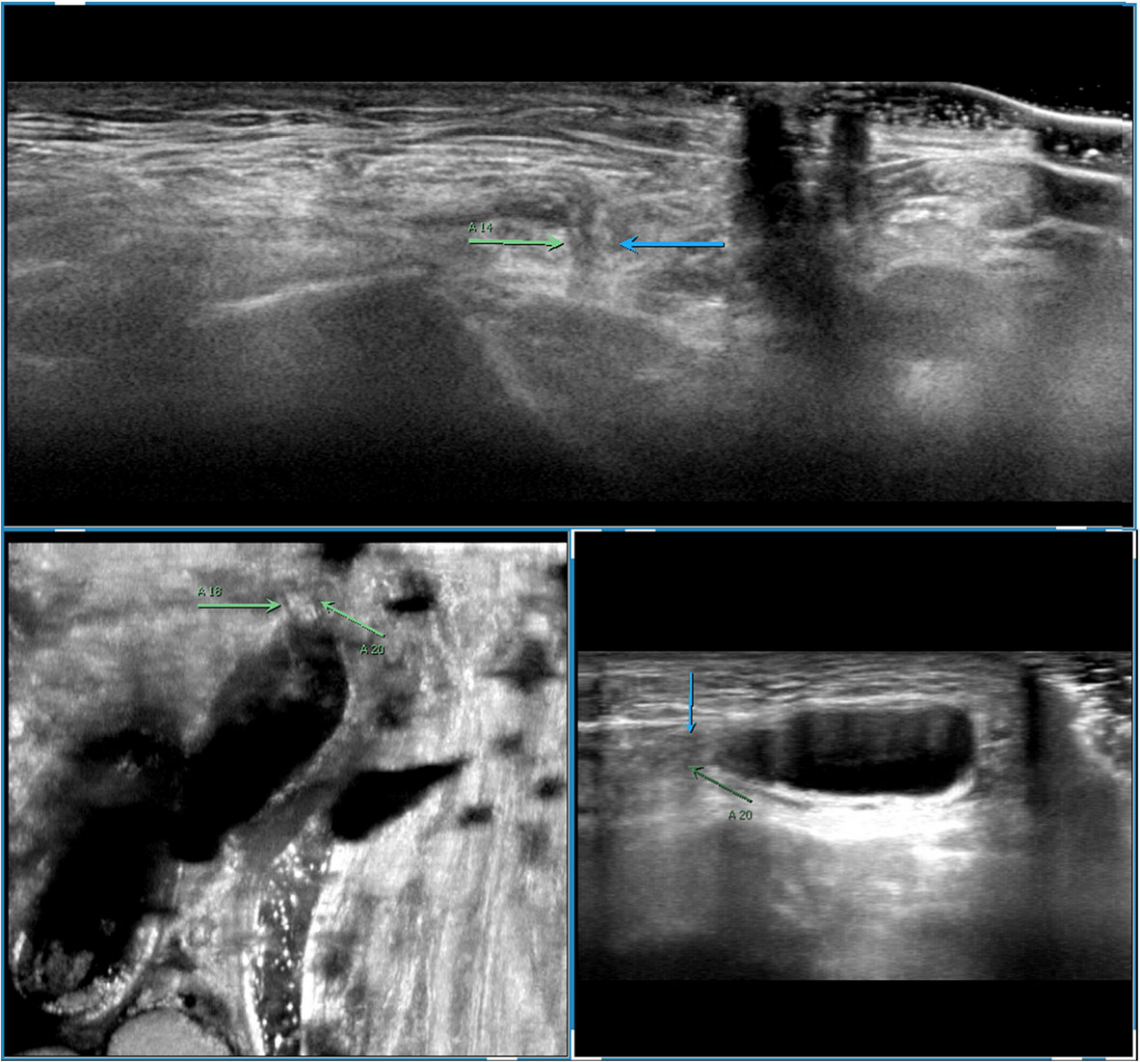
The cyst of spermatic cord showed in the ultrasonography. Arrows referred to the left spermatic cord

**Fig. 7:**
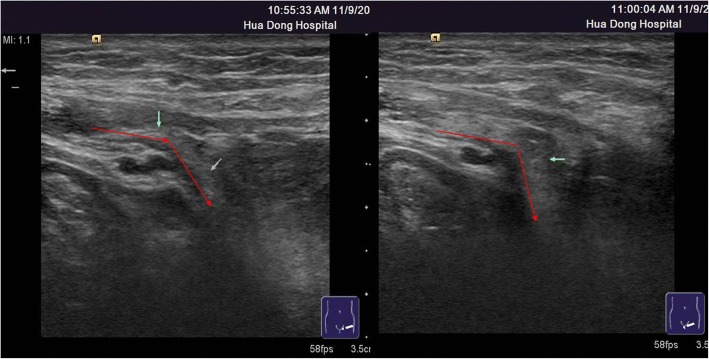
The spermatic cord showed in the ultrasonography. The spermatic cord was relaxed in supine position(the left graphy). After the patient changed to standing position(the right graphy), the spermatic cord became tight and the bending angle of it was reduced, which indicated that the spermatic cord was adhered in the deep inguinal ring

**Fig. 8:**
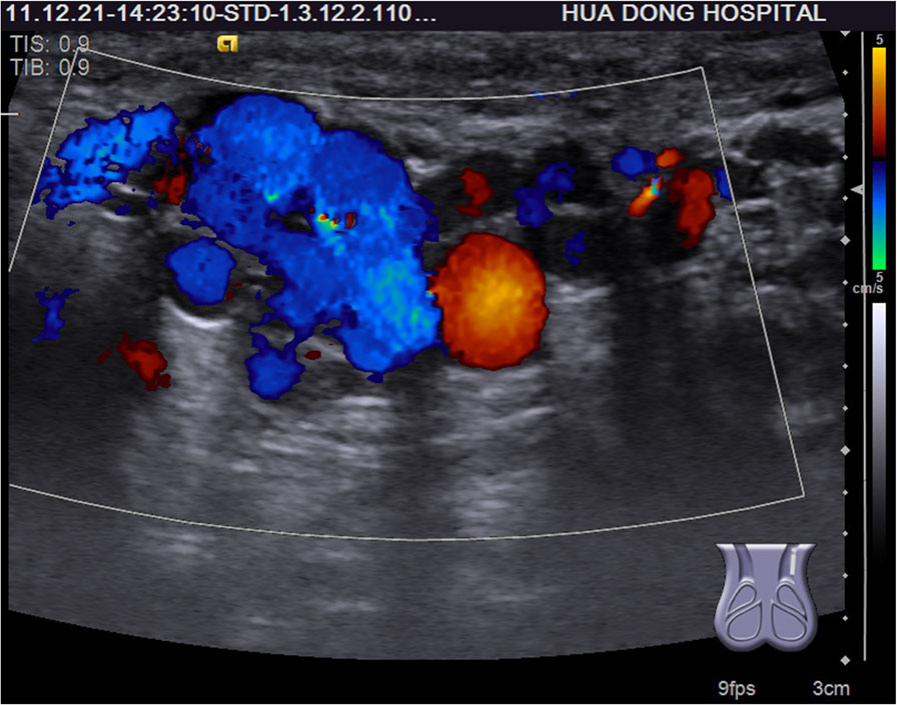
The varicocele showed in the ultrasonography

Among 121 sites with positive ultrasonic findings, 49 sites had a single positive diagnosis, 49 sites had 2 positive diagnoses and 23 sites had 3 positive diagnoses, which resulted in a total of 216 positive diagnoses: 24 cases of encapsulated effusion (11.1%), of which 14 cases were confirmed by ultrasound guided aspiration to be seroma (Fig. 1) and 10 cases were confirmed to be hematoma (Fig. 2). 59 cases of recurrent hernia (27.3%) (Fig. 3), 31 cases of shrinking mesh (14.4%) (Fig. 4), 5 cases of accumulational patch or mesh plug (2.3%) (Fig. 5), 2 cases of cyst of spermatic cord (1.0%)(Fig. 6) and 1 case of epididymal cyst (0.5%) were confirmed surgically. In addition, there appeared to be restricted motion of the spermatic cord at the reconstructed deep inguinal ring in 20 cases(9.2%)(Fig. 7), including 10 cases of the spermatic cord adhered in the deep inguinal ring, 7 cases of patch compression of the spermatic cord, and 3 cases of reconstructed deep inguinal ring stenosis, all of which were confirmed by adhesiolysis and inguinal neurotomy. Furthermore, ultrasound confirmed 19 cases of the scrotal wall edema (8.8%), 16 cases of testitis (swelling with increased blood flow) (7.4%), 16 cases of varicocele(7.4%)(Fig. 8), and 2 cases of mesh sutured into pubic tubercle(1.0%), in which the mesh and scar hyperplasia were removed surgically, as well as 5 cases of hydrocele testis (2.3%)(3 cases were confirmed by urological surgery). Ultrasonography revealed 3 cases of local weakness at the reconstructed abdominal wall, which need further confirmation by clinical follow-up.

The pain was relieved or even disappeared when patients had undergone re-operation, puncture, and other clinical operations (such as anti-inflammation therapy and traditional Chinese medicine fomentation).

### Comparison of pairwise combination of positive diagnoses

Ultrasonography revealed that 23 pain sites were found 3 positive diagnoses in each site and 49 pain sites were found 2 positive diagnoses in each site, which resulted in 167 positive diagnoses (Table 1).

**Table 1:** Pairwise comparisons of positive diagnoses

	Encapsulated effusion ①		Varicocele ②		Shrinking mesh ③		Recurrent hernia ④		Sum	χ^2^ value	*P* value
	Combined	Un- combined	Combined	Un- combined	Combined	Un- combined	Combined	Un- combined			
Encapsulated effusion	23	0	0	23	0	23	5	18	23		
Scrotal wall edema	19	0	0	19	0	19	1	18	19	①41.37	<0.05
Testitis	8	2	0	10	0	10	1	9	10		
Hydrocele testis	2	3	2	3	1	4	0	5	5		
Mesh sutured into pubic tubercle	0	2	2	0	0	2	0	2	2		
Cyst of spermatic cord / epididymal cyst	0	2	1	1	0	2	0	2	2		
Accumulational mesh	0	5	1	4	4	1	1	4	5		
Varicocele	0	16	16	0	4	12	2	14	16		
Restricted motion of spermatic cord at deep inguinal ring	0	20	13	7	8	12	8	12	20	②20.07	<0.05
Shrinking mesh	0	31	4	27	31	0	23	8	31	④13.19	<0.05
Recurrent hernia	5	29	2	32	21	13	34	0	34	③7.36	<0.05
Sum	57	110	41	126	69	98	75	92	167		

The intragroup comparisons, grouped according to pairwise combinations of 167 positive diagnoses, showed that encapsulated effusion synchronized with scrotal wall edema, varicocele synchronized with restricted motion of the spermatic cord at the reconstructed deep inguinal ring, and shrinking mesh synchronized with recurrent hernia had significant differences within the respective groups (*P* < 0.05), which occurred more frequently than other intragroup pairwise combinations.

Meanwhile, other pairwise combinations of positive diagnoses didn’t have statistically differences in the intragroup comparisons (*P* > 0.05).

There were 43 cases that postoperative chronic pain occurred, but no insignificant abnormalities were found on ultrasonography. These cases required continued follow-up.

## Discussion

Ultrasonography is a fast, effective and radiation-free examination and suitable for various kinds of positions. UVAS, as a form of advanced ultrasound technology, supplies the coronal consecutive dynamic images on the basis of the two-dimensional images, plays a complementary role to the traditional ultrasound and provides the most intuitive visual plane for clinicians to work out further surgical plans [10]. Ultrasonography can clearly reveal the corrugation of the mesh, the motion of the spermatic cord and the continuity of the peritoneum, which is conducive to diagnosing the cause of the chronic pain after inguinal herniorrhaphy [11]and an effective method of avoiding re-operation in the majority of cases.

There are two main steps of anterior open inguinal herniorrhaphy. One is separation of the hernial sac and the spermatic cord, and the other is to reconstruct the back wall of inguinal canal by suturing or patching an artificial mesh [12]. Ultrasonography shows the morphology and size of the mesh as well as the continuity of the peritoneal peritoneum, which can be used to detect the recurrent hernia. According to the literature, the chronic pain following inguinal herniorrhaphy is mainly from somatic, neurological, or visceral sources [13, 14]. Cunningham et al. [15] found that the most common type of postoperative chronic pain was somatic pain, which was mainly associated with pubic tubercle injury caused by suturing the ligament and other tissues into the pubic tubercle. Visceral pain mostly originates from the reproductive system. Ultrasonography can find that the motion of the spermatic cord decreased significantly, the flow of the spermatic vein is obstructed and the spermatic vein is expansive. Ultrasonography can also accurately diagnose the varicocele when the blood flow spectrum of varicose vein was obviously reversed in Valsalva test. Butler et al. [16] reported that the torsion and postoperative stenosis of the spermatic cord caused by scar hyperplasia, which might lead to ejaculatory dysfunction or ejaculation pain. Wantz et al. [13, 17] found that the incidence of ischemic testitis following inguinal herniorrhaphy was 0.61%, which associated with the compression caused by oversized mesh plug or an excessively small patch hole to the spermatic cord. The intraoperative damage or the ligation of spermatic arteries and veins resulted in the obstruction of venous flux and the ischemia of the testis.

Be careful to avoid the sensory nerves when the mesh is being sewed. Neuropathic pain may be due to the inguinal or genitofemoral nerves injury [18]. Ultrasonography has limitation in scanning the nerve branches. This might be the reason for the 43 cases in this study in which postoperative chronic pain was present but no significant abnormalities were apparent on ultrasonography. Caliskan et al. [19] sought the causes of pain by mean of relieving the pain by inguinal neurotomy. Wijsmuller et al. [20] determined the possibly damaged nerves through intraoperative identification and protection of the ilioinguinal nerve and genitofemoral nerve genital branch. Surgical nerve injury might cause the formation of neuromas or complete transection of nerve trunk, while postoperative adhesion and local inflammation might cause scars implanting into the nerve [21, 22].

Therefore, following guidelines, standard operation and normative process are the key to reduce the chronic pain and the other postoperative complications after inguinal herniorrhaphy.

## Conclusions

Both UVAS and traditional ultrasonography have important value in the diagnosis of the chronic pain following anterior open inguinal herniorrhaphy. Some causes of the chronic pain always occur simultaneously which should be comprehensively considered by clinical doctors. Therefore, it is necessary to conside ultrasound technology as an objective basis for the assessment of postoperative chronic pain.

## Abbreviation

UVAS: Ultrasonic volume auto-scan
